# The Potential Clinical Relevance of Necrosis–Necroptosis Pathways for Hypoxic–Ischaemic Encephalopathy

**DOI:** 10.3390/cells14241984

**Published:** 2025-12-14

**Authors:** Benjamin A. Lear, Alice J. McDouall, Olivia J. Lear, Simerdeep K. Dhillon, Christopher A. Lear, Frances J. Northington, Laura Bennet, Alistair J. Gunn

**Affiliations:** 1 Department of Physiology, The University of Auckland, Auckland 1010, New Zealand; b.lear@auckland.ac.nz (B.A.L.); a.mcdouall@auckland.ac.nz (A.J.M.); olivia.mills@auckland.ac.nz (O.J.L.); s.dhillon@auckland.ac.nz (S.K.D.); christopher.lear@auckland.ac.nz (C.A.L.); l.bennet@auckland.ac.nz (L.B.); 2 Division of Neonatology, Department of Pediatrics, Johns Hopkins University School of Medicine, Baltimore, MD 21287, USA; frances@jhmi.edu

**Keywords:** necroptosis, cell death pathways, periventricular leukomalacia, cystic white matter injury, neonatal brain injury, hypoxic–ischaemic encephalopathy, asphyxia, therapeutic hypothermia, neuroinflammation, microglia

## Abstract

**Highlights:**

**What are the main findings?**

1. Necroptosis makes a major contribution to both early and delayed brain injury after perinatal hypoxia-ischaemia.
2. Necroptotic signalling starts soon after hypoxia–ischaemia, remains active into the tertiary phase and is only partly suppressed by hypothermia.

**What are the implication of the main findings?**

1. Necroptosis and its upstream inflammatory drivers are potentially viable targets for adjunct therapies.
2. Recognising necroptosis as a key pathway suggests new approaches to alleviate cell death in future neuroprotective trials.

**Abstract:**

Hypoxic–ischaemic encephalopathy (HIE) is a major cause of neonatal brain injury and is associated with a high rate of death and lifelong disability. Its pathogenesis is still poorly understood, and there is no proven treatment for preterm infants. Therapeutic hypothermia for term and near-term infants partially improves outcomes, highlighting the need to target additional mechanisms. This review evaluates evidence that necrosis and necroptosis contribute materially to evolving brain injury in both term and preterm brains. Serial imaging studies suggest that lesions typically develop over many days after birth for term infants and over many weeks after birth for preterm infants. Growing evidence from animal studies shows that severe white matter injury can be mediated by programmed necroptosis. In particular, lesions that evolve late after acute HI are characterised by necrosis in association with agglomerations of microglia, with little apoptotic cell death. Critically, preclinical studies in large and small animals show that outcomes can be dramatically improved by very delayed intervention after HI including with cell therapy, anti-inflammatory agents, and endogenous neurotrophins. These findings strongly support the hypothesis that there may be a window of therapeutic opportunity for days or even weeks after birth to prevent delayed necrotic lesions.

## 1. Background

Neonatal encephalopathy (NE) remains a major global health problem. Hypoxia–ischaemia (HI) accounts for a substantial proportion of cases and contributes significantly to both infant mortality and long-term neurodevelopmental disability. HI represents the underlying pathophysiological insult, while the clinical manifestations are described as hypoxic–ischaemic encephalopathy (HIE). In 2010, intrapartum HI was estimated to account for approximately 1.15 million cases of NE worldwide in term and preterm infants [1]. Of these, an estimated 287,000 infants died, 233,000 survived with moderate to severe disability, and 181,000 survived with mild disability. In the United States, data from a large cohort of 115,502 preterm births suggest that HIE occurs in approximately 35 to 37.3 per 1000 preterm live births [2]. However, smaller studies using strict diagnostic criteria have reported lower rates, ranging from 5 to 9 per 1000 live births [3,4,5]. The rate of HIE in term infants is estimated to be 1–3 per 1000 live births in high-income countries and 10–20 per 1000 live births in low- and middle-income countries [6,7,8,9].

**Figure 1:**
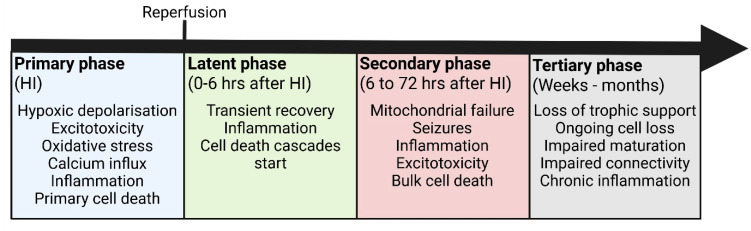
Basic schematic of the phases of HI injury. HI: Hypoxia–ischaemia. Created in BioRender.com.

The current understanding for how HI ultimately leads to brain injury is heavily reliant on animal models, many focussed on animals at human term equivalent. The evolution of injury in preterm infants is less clearly defined, largely because of the heterogeneity of injury in this population [10,11,12,13,14]. Nevertheless, it is well established that, in general, HI brain injury evolves over several pathophysiological-characterised phases (primary, latent, secondary, and tertiary phases of injury, Figure 1) as extensively reviewed elsewhere [15,16,17,18,19,20,21,22,23,24]. A basic schematic of these phases is presented in Figure 1. Each phase is associated with distinct cellular and molecular mechanisms of injury, among which necrosis and necroptosis have emerged as key contributors to neuronal cell death, particularly during the secondary and tertiary phases [25,26].

## 2. Necrosis Versus Necroptosis

Necrotic cell death is well documented in both neonatal rodents after HI and in post-mortem brains from infants with HIE. It appears to be a major contributor to early cell loss and secondary inflammatory exacerbation [25,27]. Necrosis is best understood as the morphological end-stage of uncontrolled cell death with rupture of the cell membrane, resulting in the release of pro-inflammatory cellular contents, leading to further tissue damage [28]. The process previously termed oncosis (now recommended by the Nomenclature Committee on Cell Death to be referred to as accidental cell death) describes the classical morphological features that result in necrotic cell death, including cell and organelle swelling, vacuolation, karyolysis, and the eventual cell lysis [28,29]. Accidental cell death occurs during acute HI or stroke and so has also been termed ischemic cell death. This includes depletion of energy stores and the resultant failure of ionic pumps, resulting in cytotoxic oedema and ultimately rupture [29].

Necroptosis, by contrast, is a regulated, caspase-independent form of programmed necrosis. Interestingly, necroptosis and apoptosis share many molecular components, and both are initiated by activation of membrane-bound death receptors via tumour necrosis factor (TNF)-family cytokines [30,31]. Typically, apoptosis predominates, thus necroptosis only develops when the apoptotic pathway, particularly caspase-8, is inhibited or otherwise compromised. TNF-family death receptor signalling under conditions of caspase-8 inhibition, drives receptor-interacting protein 1/3 (RIP1/RIP3) necrosome formation and Mixed Lineage Kinase Domain-Like protein (MLKL)-mediated membrane permeabilization, with subsequent release of damage-associated molecular patterns [32,33]. In the context of HIE, necroptosis has been most clearly implicated in animal models, where pharmacological inhibition of RIP1 with Necrostatin attenuates brain injury and inflammation [32,34,35]. In human neonates, evidence of necroptosis is currently limited to indirect extrapolation from expression of necroptotic mediators in human adult brain diseases, such as stroke and traumatic brain injury [36,37,38]. Beyond RIP1 inhibition, downstream components of the necroptotic pathway can also be targeted. For example, Necrosulfonamide has been shown in human cell cultures to covalently modify MLKL and prevent its membrane recruitment thus blocking necroptotic membrane rupture [39]. Obviously, necrosis and necroptosis only reflect a part of the complexity of HI brain injury, because there is a wide repertoire of regulated death pathways, whose relative contribution is shaped by developmental stage, metabolic state, and inflammation.

## 3. Other Important Cell Death Pathways

In recent years, it has become increasingly clear that there are many pathways to cell death, as listed by the Nomenclature Committee of Cell Death [40]. Of course, necrosis and apoptosis were identified early and therefore remain the most widely studied forms of cell death in neonatal HIE, largely because they are familiar and comparatively straightforward to measure. Although the growing list of cell-death subtypes may appear, at first, to be an exercise in over-classification that forces reluctant scientists to engage with more complex molecular cell biology, these distinctions are necessary. They allow us to begin explaining why injury can evolve to different severities and why the timing of pathology after HIE is so dynamic. It also makes intuitive sense that multiple avenues to cell death exist as cells are not solely injured by hypoxia, ischemia, acidosis, and inflammation, and that the impact of injury is also modulated by factors such as redox state, lysosomal and mitochondrial stability, and membrane lipid composition. Thus, these ‘newer’ pathways offer insight into aspects of injury progression that cannot be accounted for by apoptosis and necrosis alone and expand the range of potential therapeutic opportunities. To date, no pharmacological treatment has consistently and significantly improved outcomes in neonatal HIE, suggesting that previous strategies may not have targeted the right mechanisms at the right time. A more precise understanding of when and how distinct cell death pathways are engaged may therefore improve the effectiveness of future interventions across a wider range of injury severities and stages of injury evolution.

In neonatal brain injury research, animal models have been instrumental in investigating these newer regulated cell death pathways, such as the Rice–Vannucci model [41]. Many of the ‘newer’ pathways have been implicated in HIE, each likely engaged under distinct cellular conditions and within certain spatial and temporal domains of HI injury, as follows.

- Anoikis is a distinct intrinsic apoptosis triggered by loss of integrin-mediated cell anchorage and has been demonstrated in neonatal rodent HI models [42,43,44]. It is thought to contribute to neuronal death when extracellular matrix connections are disrupted, although this remains to be confirmed in human neonatal brain injury.
- Autophagy-dependent cell death is supported by evidence from multiple animal models and observations in human infants [45,46,47,48]. This form of self-digestion-related death typically arises in the secondary phase and is marked by accumulation of autophagosomes and impaired autophagic flux in vulnerable neurons [49].
- Autosis, described as cell death caused by excessive autophagy of organelles, has also been identified in cell culture and neonatal rats after HI [50]. It was recently confirmed in rodent HI models with similar autotic cell death signatures to those observed in human neonates with HIE [51].
- Intrinsic and extrinsic apoptosis remain the most widely studied pathways in neonatal HIE, with numerous rodent studies showing that blocking key apoptotic proteins attenuates injury [52,53]. Apoptotic cell death is well established in human autopsy tissue after HIE [54].
- Ferroptosis, an iron-catalysed, lipid peroxidation-driven cell death, has been shown to contribute to hippocampal neuron injury in neonatal rodent HI models [55,56]. Although direct demonstration of ferroptosis in human HIE is limited, ferroptosis-associated markers like iron accumulation are apparent in infant brains measured with MRI [57].
- Lysosome-dependent cell death, involving lysosomal membrane permeabilization and release of proteases, is suggested to exacerbate HI brain injury in rodent studies [58]. Neonatal HI disrupts neural lysosomal integrity and protein distribution [58], which can amplify cellular injury, although there is still no clear evidence of this mechanism in human neonates.
- Mitochondrial permeability transition (MPT)-driven necrosis is another injury mechanism reported in rodent and piglet studies of neonatal HI [59,60,61,62,63]. Excessive opening of the mitochondrial permeability pore during the secondary energy failure leads to energy collapse and necrotic cell death; this has made mitochondria a therapeutic target, but interventions like cyclosporine (an MPT blocker) have had mixed success in improving outcomes [60,62].
- NETotic cell death is another recently described cell death pathway wherein neutrophils release extracellular traps (NETs) that can kill cells and propagate inflammation. Initially recognised in animal studies of neonatal infection [64], NETosis has more recently been observed after neonatal HI in rodents even during therapeutic hypothermia [65], indicating that neutrophil infiltration and NET release in the injured brain may sustain inflammation. Importantly, potentially this may be a part of why cooling alone is not fully protective.
- Parthanatos is a distinct, caspase-independent death pathway triggered by intense metabolic stress that stimulates poly(ADP-ribose) polymerase-1 (PARP-1), leading to the release of apoptosis-inducing factor (AIF) from mitochondria [66]. This mechanism causes a form of cell death resembling necrosis and can occur rapidly after HI [67]. However, despite early evidence of parthanatos-mediated neuronal death in neonatal HI, neuroprotective strategies targeting parthanatos have had inconsistent effects [68].
- Pyroptosis is an inflammatory form of programmed cell death governed by the NLRP3 inflammasome and executed by caspase-1. It has been demonstrated in neonatal rodents after HI [69,70]. Further, pyroptosis-associated markers have been consistently detected in the brains of human infants dying with HIE [71].

## 4. Accidental Necrosis Is the Primary Cell Death Pathway During Hypoxia–Ischemia

The period of HI itself may be termed the primary phase. At the cellular level, a combination of insufficient oxygen and metabolites impairs the production of ATP. Without sufficient ATP production, intracellular homeostasis becomes compromised as ATP-dependent Na^+^/K^+^ pumps fail [18,20,72]. Eventually, cells depolarise, which increases the intracellular concentration of Na^+^, Ca^2+^, glutamate, and water, with a reciprocal loss of K^+^ [18,72,73]. This anoxic depolarisation triggers waves of depolarisation to neighbouring cells [73]. A prolonged period of anoxic depolarisation can cause cellular injury [18,74]. In preterm fetal sheep for example, prolonged asphyxia causes a delayed increase in cortical impedance, a measure of cell swelling, starting 5 to 6 min after the start of profound HI [75].

Importantly, the altered ionic gradient across the cellular membrane has detrimental effects on cells by activating multiple deleterious pathways [74]. The presence of high [Ca^2+^] activates multiple enzymes such as phosphatases, endonucleases, lipases, and proteases, which trigger the generation of reactive oxygen and nitrogen species [72,76]. These reactive species contribute to oxidative stress, lipid peroxidation, and mitochondrial dysfunction, further exacerbating cell injury [72,76]. Furthermore, the influx of calcium activates neuronal nitric oxide synthase (nNOS), an enzyme that converts L-arginine to citrulline and nitric oxide (NO) [77,78]. NO can then react with superoxide to produce peroxynitrite, a highly reactive molecule that initiates lipid peroxidation and cytotoxic events [78,79]. This oxidative stress plays a significant role in cellular loss during the primary phase and potentially facilitates future programmed cell death [80].

Of course, if a period of severe HI persists for long enough, excessive water will enter the cell, leading to severe cell oedema, followed by necrosis [74]. This is the main form of cell death in the primary phase. In a study investigating the maturational difference in the response to umbilical cord occlusion (UCO) in 0.6, 0.7 and 0.85 gestation fetal sheep, metabolic failure, measured with near-infrared spectroscopy (NIRS) showed a profound rise in cytochrome oxidase (CytOx) between 5 and 13 min during the UCO; this rise is greater than 0.6 gestation, but less than in 0.85 gestation fetal sheep [75]. This difference between gestations demonstrates the more effective adaptive hypometabolism at younger gestations likely due to reduced axonal complexity [75]. Furthermore, at 0.7 gestation, cytotoxic oedema measured by brain impedance also showed a gradual increase starting roughly 8–10 min during UCO, demonstrating that after the onset of anoxic depolarisation it progresses leading to cell swelling [75]. However, despite this, after reperfusion the vast majority of neurons and glial cells survive [81].

## 5. Apoptosis and Necrosis During the Secondary Phase After Hypoxia–Ischemia

About 6–15 h after HI, magnetic resonance spectroscopy in newborn piglets (equivalent to a preterm human) showed a secondary deterioration, termed the secondary phase, as shown by delayed loss of oxidative metabolism and ultimately extensive cell death [12,13]. Similarly, in near-term fetal sheep this phase is characterised by delayed onset of seizure activity, and cortical cytotoxic oedema [82]. Continuing functional suppression, increased cerebral oxygenation and loss of CytOx or phosphates and lactate in the secondary phase denote that this reflects mitochondrial failure [12,13,83,84]. The mitochondrial failure and subsequent neurodegeneration observed in this phase of injury is likely a culmination of various factors such as energy supply depletion, mitochondrial damage, NMDA receptor activation, excitatory amino acids, cellular calcium overload, reactive oxygen species, pro-apoptotic and inflammatory proteins and trophic factor withdrawal [18,25,59,80,85]. Clinically, infants with HIE exhibit a loss of energy metabolism in a similar time frame, characterised by a reduction in phosphocreatine and an increase in lactate [84,85,86].

It is important to note that during the secondary phase of injury there is no single exclusive cell death pathway [87,88]. Rather, necrotic and apoptotic pathways co-exist, with other regulated cell death pathways, resulting in a phenomenon known as the “apoptotic continuum” where a cell can display elements of both apoptosis and necrosis at the same time with one or the other being more prominent, depending on maturation, the nature of the insult and regional severity [87,88,89,90]. Recent studies also show that autophagy–lysosomal and mitophagy pathways are activated during the secondary phase, with increased autophagy associated proteins [26]. Nevertheless, within the first 24 h after HI, extensive evidence from human post-mortem studies and experimental models demonstrate cell death involving both necrotic and apoptotic components [59,90,91,92,93,94,95,96,97]. Northington described in neonatal rodents that neurons in the forebrain showed classic necrosis features, such as swelling, vacuolation of organelles and loss of membrane integrity, as well as apoptotic features, such as shrunken cytoplasm, and compact chromatin [25]. More recently, newer multiparameter microscopy and flow-cytometry techniques that assess cell and nuclear morphology, annexin-V/propidium iodide staining, DNA fragmentation [TUNEL] and caspase activation have been developed [98]. These newer histological techniques provide a framework to distinguish predominantly apoptosis from necrosis-like death and identify cells with overlapping features along the apoptosis–necrosis continuum.

## 6. Is This Early Form of Necrosis, Necroptosis?

A key question is whether the necrotic component of cell death in the secondary phase is mainly due to passive necrosis or regulated necroptosis. Experimental data now indicate that at least a proportion of this morphologically necrotic cell death is mediated by necroptotic pathways. During the secondary phase there is at least some oligodendrocyte precursor cell death that is morphologically necrotic after severe HI [96,99]. Pre-oligodendrocyte cell culture studies suggest that neuroinflammation may increase phospholipase activity, which induces the production of arachidonic acid, which in turn is metabolised into reactive oxygen species and peroxides, thus increasing oxidative stress [100,101]. This is important as pre-oligodendrocytes are vulnerable to oxidative stress. Interestingly RIP1 plays a key executioner role in this situation as oxidative stress induced by arachidonic acid or glutathione (an important antioxidant) depletion may initiate necroptosis without TNF receptor signalling [101]. This is supposedly possible if the redox state of a cell shifts with a reduction in glutathione, causing RIP1 to dissociate from the TNF receptor complex, therefore allowing RIP1 to initiate necroptosis [101]. Importantly, RIP1 blockade with Nec-1 treatment prevented arachidonic acid-induced necroptosis. Taken together, these data suggest that the oxidative and inflammatory milieu generated by HI promotes RIP1-dependent necroptotic cascades in vulnerable pre-oligodendrocytes early in the secondary phase, rather than purely passive necrotic cell death.

Experimentally, necroptosis in HI tissue can be quantified with the expression and phosphorylation of RIP3 and MLKL (pMLKL) as well as membrane rupture shown by propidium iodide uptake or HMGB1 release [98,102,103]. Currently, no circulating biomarker of necroptosis has been validated for neonatal HI brain injury. Serum assays may not be able to distinguish brain-derived necroptosis from parallel activation in other organs secondary to global HI. Nevertheless, there is evidence of higher baseline and rising RIP3 levels over the first 24–72 h in sepsis or trauma in critically ill adults, with elevated levels of HMGB1 and that MLKL closely correlates with RIP3 concentrations, organ failure and death [104,105,106,107].

While it is clear that early cell death after HI includes a substantial component of accidental necrosis, the precise timing and distribution of necroptosis in vivo remain uncertain. In near-term mice, morphologically necrotic neuronal death is prominent in the cortex and striatum at 3 h after HI, followed by a shift towards predominantly apoptotic cell death within the thalamus and brainstem at 24 h post HI [108,109]. Consistent with these rodent data, the process of striatum neuronal degeneration in the term piglet is primarily characterised by necrosis within 3 h post HI and necrotic injury was apparent up to 24 h accompanied by reduced levels of glutathione and increased oxidative stress [110]. In an early trial, Nec-1 administered 15 min after HI in P7 mice failed to reduce neuronal loss in the hippocampus, cerebral cortex or thalamus during the first 24 h [89], suggesting that the very early necrotic neuronal death in this model is not primarily mediated by RIP1-dependent programmed necrosis.

In contrast, recent studies implicate necroptotic signalling in the secondary phase of injury. Administration of Nec-1 before and immediately after hypoxia in P12 rats reduced cortical and striatal pMLKL and TNF-α at 5 h post HI and, when combined with hypothermia, led to reduced loss of ipsilateral hemisphere, caudate–putamen and hippocampal volumes on MRI at P20–21 [103]. Furthermore, administering a microRNA (miR-105) 6 h after HI in P10 rats suppressed both apoptosis and necroptosis, as shown by reduced expression of apoptosis and necroptosis-associated proteins, smaller infarcts at 24 to 48 h, and improved long-term cognitive performance [111]. Taken together, these data suggest that early cell death after HI is a mixture of necrosis and necroptosis. The very earliest wave of morphologically necrotic neurons within the first few hours after HI is unlikely to represent pure RIP1-dependent necroptosis, but necroptotic signalling is clearly engaged within the evolving secondary phase and contributes to subsequent tissue loss.

## 7. Tertiary Phase and Necroptosis

As the injury progresses from the secondary into the tertiary phase, the brain is thought to initiate repair and remodelling processes, as indicated by increased cell proliferation observed from around three days post injury in preclinical models [112,113,114]. However, a range of ongoing deleterious mechanisms, established during the secondary phase cause long-term impairment of brain development, leading to disrupted cell maturation, connectivity and overall brain growth [115,116,117]. The most severe manifestation of HI injury is cystic injury, which is characterised by foci of necrosis involving all cellular elements within the lesion area [117].

In preterm infants, cystic lesions are commonly observed in the regions identified by Banker and Larroche, situated in the deep white matter adjacent to the ventricular wall and can also extend into the centrum semiovale and subcortical white matter [117,118]. Microscopic lesions, typically measuring less than 1–2 mm, are more common but are easily missed on ultrasound; however, these lesions can progress over time and manifest as macroscopic lesions, exceeding 2 mm in size [118]. Although rates of cystic WMI have gradually fallen [119], cystic WMI remains highly associated with cerebral palsy and is seen in 1–3% of preterm born babies [119,120,121].

Importantly, cystic brain lesions develop over many weeks. The typical ultrasonographic appearance of cystic WMI is of cerebral echolucencies, consistent with cysts, often accompanied by ventricular enlargement [122] and is most commonly detected around 4 weeks after birth [122]. In a large serial ultrasound study of preterm infants, most cystic lesions were absent on early images and then were present several weeks after birth, at a mean detection time of 36 weeks post-menstrual age [122,123]. In another ultrasound study, cystic lesions were identified in over half the recruited infants at one month. These lesions then resolve by term equivalent age [122]. These findings indicated that a significant amount of injury occurs after the first 3 days of life. This pattern of injury has not been reported in most pre-clinical animal studies. The mechanism responsible for such delayed development of significant brain injury is yet fully elucidated. Speculatively, it is possible that necroptosis is initiated early, around the time of birth in preterm infants, but then takes roughly 2–3 weeks to manifest, eventually resulting in the thinning, and eventual, liquefaction of tissue as seen within a cystic injury [117,124].

Very few studies have investigated necroptosis weeks after injury in the perinatal brain. In preterm fetal sheep recovered to 14 days after 25 min of UCO 3/5 animals displayed small patches of necrotic cells in the subcortical and PVWM [125]. In another preterm fetal sheep study, which used serial put-downs, fetal brains were macroscopically normal 3 and 7 days after 25 min of UCO, but by 14 and 21 days, showed widespread tertiary cell loss, with cystic lesions predominantly in the temporal white matter, accompanied by white matter atrophy and ventriculomegaly [23].

In P7 rats, cystic cerebral infarctions have been observed at 2 weeks after severe HI with no further evolution after 4 and 8 weeks recovery from HI [126]. By contrast, in the same study, moderate HI was associated with no significant infarction at 2 weeks recovery, but a delayed appearance of extensive cortical infarction by 8 weeks recovery, which was comparable to the extent observed in the severe HI group [126]. This study illustrates that the timing and trajectory of perinatal brain injury is modulated by the severity of HI. Unfortunately, it did not investigate the type of cell death involved or whether moderate and severe HI triggered different cell death pathways. For now, it is still unclear whether tertiary cystic lesions reflect a single, early commitment to necroptosis with very delayed execution, or repeated re-stimulation of necroptotic pathways over several weeks after HI.

## 8. When Is Late Necroptosis Initiated?

Studies directly investigating late programmed necrosis in perinatal HI models have involved near-term equivalent mice and, more recently, preterm fetal sheep. Although, Northington et al. found that Nec-1 did not provide neuroprotection against early evolving injury, it did show long-term protection, preventing delayed neuronal injury out to 21 days after HI [89]. Importantly, in these neonatal rodent studies only a single Nec-1 infusion was given at 15 min post-HI [89]. With a half-life of 90 min, this indicates that in this experimental setting, the biochemical cascades leading to delayed programmed necrosis must have been initiated very early during the first hours of recovery after HI, and yet also indicates that these cascades took many weeks for programmed necrosis to be finally executed. In another rodent experiment that had a much shorter recovery period, Sun et al. induced brain injury at P9 and administered Nec-1s once daily for 5 days; at 5 days after surgery they reported reduced RIP1/RIP3/MLKL signalling and fewer necrosis-like cells. Importantly, this histological improvement was associated with improved behavioural performance at P45. This in-depth study provides acute and long-term functional characterisation, although the long-term histological changes remain to be seen [127].

Complementing these findings, in chronically instrumented preterm fetal sheep at 0.7 gestation, Nec-1s given at 3, 8 and 13 days after 25 min of UCO was associated after 21 days recovery with a marked reduction in cystic white matter injury in the temporal lobe [35]. Although the programmed necrosis cascade was clearly initiated early after HI in these studies, it is not clear when cells were irreversibly committed to necroptotic cell death. Anti-inflammatory treatment studies that more broadly interact with upstream determinants of necroptosis have shown robust protection when started 3 days post injury, suggesting that the necroptotic cascade has been initiated by this timepoint, but is still modifiable during the tertiary phase [21,128].

**Figure 2:**
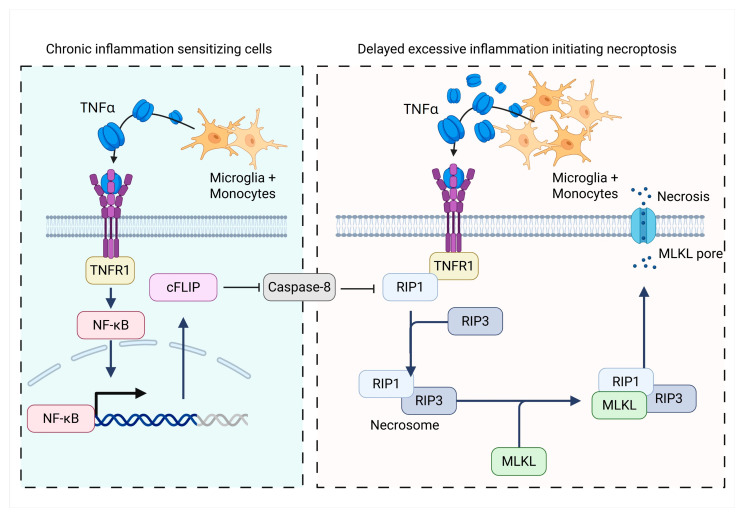
Hypothesised mechanisms mediating necroptosis after HI. Chronic inflammation after HI increases NF-κB–driven cFLIP expression, inhibiting caspase-8 and permits RIP1–RIP3 necrosome formation, committing cells to necroptosis. Created in Biorender.com.

For necroptosis to proceed, the apoptosis cascades must be inhibited. Biochemically this is because caspase-8 cleaves RIP1 and RIP3 complex and thus prevents them interacting to form the necrosome [39,129]. This relationship has been demonstrated when caspase-8 is pharmacologically inhibited, triggering necroptosis which in turn can be prevented with Nec-1s treatment. Caspase-8 inhibition has been implicated in multiple sclerosis where cortical lesions showed reduced caspase-8 activity and an increase in cFLIP, (an inhibitor of caspase-8) [130,131]. It was suggested that caspase-8 protein expression within a cell does not fluctuate, allowing small variations in cFLIP levels to sensitise the cell to necroptosis [132]. Importantly, cFLIP is tightly controlled by nuclear factor (NF)-κB and HI has been shown to cause a three-fold increase in NF-κB in mice 24 h after HI, likely induced by pro-inflammatory cytokines like TNF [89,132,133]. Therefore, an increase in TNF may impair caspase-8 activity and subsequently sensitise cells to necroptosis. Nec-1 in turn has been shown to reverse this by decreasing NF-κB and FLIP gene and protein expression, as well as reduce pro-inflammatory cytokines [89]. Again, this suggests that exuberant TNF would also be expected to stimulate the extrinsic apoptotic pathway [88]. See Figure 2 for a summary of the mechanisms hypothesised to mediate necroptosis.

Microglia are a major source of pro-inflammatory cytokines, particularly TNF [134]. Microglial activation occurs early after HI and, unsurprisingly, increased numbers of microglia are observed in WMI [23]. Consistent with this, microglial proliferation, mediated by TLR-family activation [135], has been shown to occur by 20 min after HI with microglial numbers increasing substantially over the first 48 h post HI [136,137]. Activated microglia can directly induce an increase in the permeability of the blood–brain barrier [138], potentially resulting in an infiltration of peripheral macrophages, which may further exacerbate neuroinflammation [113]. These events consistently precede any detectable injury [23,134,136]. Excessive microglial activation at the time of HI may programme cells to undergo cell death, attributed to the release of various death receptor ligands by microglia, such as TNF, Fas ligand, TNF-related apoptosis-inducing ligand (TRAIL), and TNF-related weak inducer of apoptosis (TWEAK) [139]. These ligands can trigger the death-inducing signalling complex, classically leading to apoptosis through caspase-8 and caspase-3 activation [85,140]. However, experimental work in ischemic brain models now shows that death-receptor signalling in a caspase-8 inhibited context also drives RIP3/MLKL-dependent necroptosis, and that this necroptotic signalling in neurons feeds back to shape microglial phenotype [141].

Traditionally, microglia were classified into pro-inflammatory ‘M1’ and anti-inflammatory ‘M2’ phenotypes [142]. However, it is now acknowledged that microglia exist in various subgroups with both anti-inflammatory and pro-inflammatory roles, making it inappropriate to label them solely as M1 or M2 [143]. The temporal changes in microglia phenotype have been investigated in a Rice-Vannucci P9 mouse study where at 24 h after HI, a significant expansion of CD86^+^ M1 (pro-inflammatory) microglial cells was observed, while the proportions of CD206^+^ M2 (anti-inflammatory) microglial cells and cells lacking both markers were reduced [144]. By 7 days after HI, the proportion of CD86^+^ cells steadily decreased over time, but their total numbers remained elevated and always outnumbered CD206^+^ M2 cells, which peaked between 5 and 7 days before falling [144]. Recently, a similar Rice Vannucci study using P3 mice showed a transient rise in CD86^+^ cells and a fall in CD206^+^ at 14 days [145]. Furthermore, this study explored cytokines measured with enzyme-linked immunosorbent assay and found a gradual increase in periventricular IL-1β mRNA as well as a major increase in TNF mRNA, both peaking at 14 days post-HI [145].

Complementary work in the cortex of adult mice after cerebral ischemia shows that neuronal necroptosis can shift microglial phenotypes, such that genetic deletion of RIP3 or MLKL reduces expression of M1 associated markers like iNOS, TNF, IL-12 and IL-18 and increases Arg-1, IL-4 and IL-10, while conditioned medium from necroptotic neurons drives an increase in M1 phenotypes [141]. Furthermore, macrophage studies indicate that classically activated M1 cells are intrinsically more susceptible to RIP3-dependent necroptosis than M0 or M2 cells, suggesting a link of the pro-inflammatory phenotype to heightened necroptotic vulnerability [146]. Taken together with studies of HI in neonatal rodents showing an early and sustained elevation of CD86^+^, TNF/IL-1β positive microglia over the first week with only a delayed rise in reparative phenotypes around 5 to 7 days [144,145], these data support that early necroptotic death of neurons (and possibly oligodendrocytes) with high levels of TNF promotes and is reinforced by M1 microglia, creating a feed-forward loop that maintains a necroptosis permissive, pro-inflammatory environment long into the tertiary phase.

## 9. Effects of Hypothermia on Cell Death Pathways

Currently, therapeutic hypothermia (TH) is the standard of care for term infants with moderate–severe HIE. Clinical trials and meta-analyses have shown that cooling initiated soon after birth significantly improves survival and long-term outcomes in high income countries [147,148]. Through extensive optimisation protocols hypothermia has been found to be most effective when administered before the secondary phase of injury begins and continued for 72 h [149,150]. Therapeutic hypothermia has wide sweeping effects that overall confers neuroprotection through the modulation of multiple injury pathways, such as inflammation, excitotoxicity, seizures and cell death as reviewed in detail in [151]. Evidence suggests that TH exerts both anti-apoptotic and anti-necrotic effects after HI. For example, in P7 rats exposed to carotid artery ligation and hypoxia, hypothermia decreased apoptosis and necrosis in the cortex and hippocampus, compared with normothermia rats 24 h after HI [152]. Similarly, in a newborn piglet model of global HI, TH was associated with a marked reduction in apoptotic cell death, although the fraction of acutely necrotic cells was not significantly changed by TH, suggesting that while hypothermia effectively attenuates apoptosis, it may have a more limited impact on the immediate, unregulated necrotic cell loss during the primary phase of injury [153].

In addition to reducing overall cell death, therapeutic hypothermia appears to shift the predominant mode of cell death under certain conditions. In a recent study investigating postmortem tissues from non-cooled infants who died with HIE, the main pathology was dominated by necrotic degeneration in vulnerable regions (hippocampal CA1 and striatum). Strikingly, brains of infants treated with hypothermia showed a shift toward apoptosis or mixed apoptosis–necrosis continuum, with fewer purely necrotic profiles [154]. By contrast, studies in neonatal piglets did not show such a shift in cell death morphology with cooling, instead hypothermia provided robust neuroprotection in piglets primarily by preventing necrotic injury, suggesting a potential inter-species difference in caspase recruitment [155].

Despite the benefits of TH, it is clear that injury processes including necrosis and necroptosis are not completely prevented by cooling as some infants still develop significant brain injury even after TH. Speculatively, this may reflect cell death cascades that continue to evolve beyond the window of TH. Experimental studies in adult and neonatal models show that TH can dampen upstream death-receptor signalling, including Fas/FasL expression, caspase-8 and caspase-3 activation, and reduce pro-inflammatory cytokines such as TNF-α, which would be expected to limit both apoptosis and the initiation of necroptosis [156,157,158,159]. However, in neonatal HI, TH only partially modulates cytokine levels. Piglet and human studies report early changes in some systemic and CSF cytokines such as lower IL-6, TNF-α, and higher IL-10, but cytokines remain elevated overall and in piglet models of perinatal HI and LPS sensitised HI, pro-inflammatory cytokines can rebound after rewarming or remain high despite TH, suggesting that the upstream inflammatory drivers are attenuated rather than abolished [160,161,162,163,164]. Consistent with this, a rodent study demonstrated that hypothermia alone had no measurable effect on the activation of necroptosis markers (such as phosphorylated MLKL) hours after HI, nor did cooling prevent long-term brain volume loss on MRI [103]. By contrast, blocking necroptosis pharmacologically with Nec-1 in combination with hypothermia provided additive neuroprotection and showed reduced expression of necroptotic effectors (such as phosphorylated MLKL and TNF-α) and significantly preserved brain tissue volumes compared to hypothermia alone. Thus, these data suggest that while TH may indirectly influence necroptosis initiation through partial suppression of caspase-8/TNF-α signalling and neuroinflammation, regulated necrotic pathways remain active under cooling and could contribute to treatment failure in a subset of infants. Consequently, targeting necroptosis could augment the neuroprotective effects of hypothermia, suggesting a strong rationale to explore combination therapies, such as adding cell death modulators to the standard cooling regimes to further reduce HI brain injury.

## 10. Conclusions

Neonatal HI brain injury is a dynamic process involving a complex array of cell death mechanisms. Our understanding of these mechanisms has grown substantially in recent years, but the clinical relevance has not yet been established. The evidence reviewed here suggests that necroptosis and related regulated necrotic processes are likely to account for a substantial portion of the injury that persists despite cooling, and so these pathways represent promising targets for neuroprotective therapies.

## Data Availability

No new data were created for this review.

## Abbreviations

CytOx—cytochrome oxidase; FasL—Fas ligand; cFLIP—Cellular FLICE (FADD-like IL-1β-converting enzyme)-inhibitory protein; HI—hypoxia-ischaemia; HIE—hypoxic–ischaemic encephalopathy; HMGB1—high-mobility group box 1 protein; IL—interleukin; iNOS—inducible nitric oxide synthase; MLKL—mixed-lineage kinase domain-like protein; MRI—magnetic resonance imaging; NMDA—N-methyl-D-aspartate; NIRS—near-infrared spectroscopy; NE—neonatal encephalopathy; NF-κB—nuclear factor kappa B; P—postnatal day; RIP1/3—receptor-interacting protein kinase 1/3; TH—therapeutic hypothermia; TNF—tumour necrosis factor; TRAIL—TNF-related apoptosis-inducing ligand; TWEAK—TNF-related weak inducer of apoptosis; TUNEL—terminal deoxynucleotidyl transferase-mediated deoxyuridine triphosphate nick-end labelling; UCO—umbilical cord occlusion; WMI—white matter injury.
